# A Desire for Parsimony

**DOI:** 10.3390/bs3040576

**Published:** 2013-10-24

**Authors:** Lawrence J. Cookson

**Affiliations:** School of Biological Sciences, Monash University, Clayton, Vic. 3800, Australia; E-Mail: Laurie.Cookson@monash.edu

**Keywords:** parsimony, desire, wildness, music, limbic system, beauty

## Abstract

An understanding of wildness is being developed as a quality of interactive processing that increases survival opportunities in nature. A link is made between the need to improve interactive quality for wildness, and cognitive desires and interests in art, music, religion and philosophy as these can also be seen as attempts to improve interactive quality internally and externally. Interactive quality can be improved through gains in parsimony, that is, simplifications in the organisation of skills. The importance of parsimony in evolution is discussed, along with indicators of an internal parsimony desire that experiences joy if achieved through processes such as insight and understanding. A mechanism for the production and measurement of the parsimony desire is proposed, based on the number of subcortical pleasure hotspots that can be stimulated at once within the ‘archipelago’ available in the limbic system.

## 1. Introduction

A range of fundamental desires such as hunger, sex, thirst, excretion and the desire for thermal comfort are well known, common to most animal species, and meet essential survival needs. Their production can be ascribed to specific bodily structures, such as when low blood sugar and fatty acid levels stimulate the hypothalamus to signal the stomach to produce ghrelin, a hormone that triggers hunger [1,2]. A ‘higher’ set of cognitive desires, best demonstrated in our species through our interest in art, music, poetry, philosophy and religion are also known. However, it is difficult to assign these to any specific adaptive survival need, let alone identify the tissues and mechanisms responsible for their production. These intellectual desires are often seen as relics, spandrels or superfluous by-products of the more fundamental desires and their accompanying adaptations [3]. For example, Pinker [4] considers that ‘The function of the arts is almost defiantly obscure’, while Ehrlich [5] considers that the arts can be seen as aesthetically pleasing rather than functional. Discussion of this problem is especially active in research on music [6,7,8,9,10]. 

The global recorded music industry revenue was worth US$16.5 billion in 2012 [11], yet to some music is an enigma and confers no survival advantage, leading Pinker to liken it to evolutionary ‘cheesecake’, and perhaps a byproduct of the more pressing need to develop language skills [4,12]. More recent consensus is that music is adaptive, partly because of its early appearance and ubiquitous nature in our species; however, the fundamental reasons for adaptiveness have not been isolated. Music may be a form of sexual selection for attracting mates by showing that one can pursue such trivial fancies because there is so much in reserve, reflecting superior fitness [13]. Or perhaps its function is to promote social bonding and cohesion [14,15], especially between parent and infant [16], or is it a form of beneficial play [10]?

Another explanation may arise from our improved understanding of the role of wildness in nature. Achieving a state of wildness can improve survival opportunities in nature, which has been demonstrated in comparisons between captive reared animals and their wild counterparts (or at least, those reared in semi-natural habitats), where wild animals develop much better skill at predator avoidance and survival [17,18,19], and foraging and tool use [20,21]. Roche *et al*. [22] found that plovers captive reared from wild abandoned eggs fledged 56% fewer young when re-released compared to wild-born plovers. Beck *et al*. [23] considered that the re-introduction of captive-born animals into the wild had only an 11% success rate. Such large differences in performance skills are not based on genetics, but show a strong selective advantage towards those who can learn wildness as a selected form of cognitive ability.

The key to understanding the value of wildness is that it focuses upon measuring the interactive quality that an animal can have internally and externally, and has been defined as a quality of interactive processing between organism and nature where the realities of base natures are met, allowing the construction of durable systems [24]. A wild animal is more connected and honest to its environment, more automatic and intuitive in its skill base, and more likely to give fast and appropriate reactions. If prime biological importance is placed on wildness, then adaptations for improving interactions to the quality needed for wildness should occur, allowing an alternative purpose and mechanism to be suggested for the ‘frivolous’ desires. The main methods used to improve interactive quality are through parsimony and attunement [24]. Parsimony causes a synthesis of improving interactive skills because it seeks greater efficiency and broader application, while attunement ensures that there is no barrier to linking and bonding with outside things or environment as these might then further inform the construction of internal parsimony. Wildness requires that adaptations, many or few (simple or complex organisms), are efficiently tied together into the most direct arrangement available.

The value of parsimony has long been recognised in evolution when constructing phylogenetic trees. In this process, the most accurate representation of evolution is assigned to the tree with fewest steps. Parsimony is the best bloodhound tracker for selecting the pathway of evolution through the species and determining their relationships [25]. There are computer programs such as PAUP [26], PHYLIP [27] and TNT [28] that can take a maze of species and their lists of characters, to produce a tree-like diagram (cladogram) of evolution by finding the most parsimonious route. It does not always give the perfect answer [29,30], but is the best rule of thumb for determining phylogenies [31,32]. While parsimony here is being used to compare evolutionary trees, it also suggests that an organism that can become more parsimonious than another is likely to survive and progress further along the evolutionary tree. Parsimony would not impede the production of increasingly complex organisms during evolution, although it would favor complexities that can be met by fewer steps or the simplest and most efficient arrangements. Certain arthropods might gain a defensive mechanism by simply and incrementally turning their setae into barbed urticating setae that easily detach from the integument when disturbed [33]. While predators that can learn to resist toxic prey based on the simple rule ‘avoid red-colored prey’ may survive longer than another that must experience the distaste of every toxic red species encountered [34,35]. Indeed, Batesian mimicry by non-toxic species relies on predators learning such parsimonious rules [36].

Organisms that improve the parsimony within the organisation of their information and behaviour may gain an evolutionary advantage. ‘The rationale of the parsimony principle is that if organisms would develop a suboptimal information processing strategy, this would lead to a waste of metabolic energy’ [37]. Parsimony allows a solid behavioural foundation to be built, where a core set of instincts and skills can be learnt to give the adaptability and relevance needed to survive in a niche. Shortcuts, such as those found through cunning [38,39], can also improve an animal’s parsimony. A keen sense for parsimony could act like an internal sorting mechanism or redundancy detector when deciding which pathway to take.

The importance of parsimony is also supported by our understanding of the way the human brain learns. When born the human brain contains about 80 billion neurons and 150 trillion synapses [40], and within this brain the cerebral cortex may contain up to 39 billion neurons although probably fewer [41]. However, about half the synaptic connections in the cortex are lost during childhood development, and pruning continues well into the late 20’s [42,43]. Such a major reduction in the number of possible connections is consistent with the involvement of a parsimonious mechanism that reduces complication in the brain’s cognitive functions while at the same time making it more effective and capable [44]. Indeed the joy and pleasures obtained during brain interaction appear to be telling us about parsimonious achievements, and may re-enforce those shortcut pathways that lead to pleasurable or useful outcomes over those that are less informative and laboured.

## 2. Signs of Appreciation for Parsimony

The ‘higher’ desires could be re-interpreted as signs that we have a fundamental desire for the achievement of parsimony, which is adaptive due to the gains that can then be made in wildness. Numerous examples suggest that finding a new parsimony is an important joy in human thinking. This reaction, the sudden click of realisation into a simpler yet more understanding parsimony, can be expressed as a Eureka moment [45,46,47], or with the expression that the penny has dropped. The joy of finding a new insight is an example of improved parsimony in mental pathway organisation. A keen wit can also be appreciated as a source of insight that simplifies pathways. Some other obvious examples of appreciation for parsimony include our positive reactions to understanding, achievement, inspiration and realisation. These impacts improve the efficiency and breadth of how our knowledge and abilities are organised. 

Our desire for parsimony may also underpin our notions for beauty. There is the perception that naturally produced items have a greater elegance over artificial things [48], which is another way of saying they have greater simplicity in design or parsimony. Wilderness and wild animals are often seen as beautiful, and visits to national parks can provide re-evaluation and nourishment to a stressed mind [49], suggesting there is a simpler way. It may be no coincidence that often, the more one understands the more beauty that can be seen, as noted by the nineteenth century painter Camille Pissarro [50]. The early philosopher Francis Hutcheson also considered that beauty could be perceived by an internal sense for uniformity amidst variety [51], *i.e.*, a sense for parsimony.

Further examples that identify our appreciation and desire for parsimony include our preference for symmetry, which allows the same vision to be seen using fewer rules. For example, there have been many studies into what makes the human face seem beautiful, with symmetry being one of the most important factors [52]. Newborn infants have a preference for attractive faces, suggesting an innate desire rather than cultural learning for what is beautiful [53], which could arise from our desire and sense for parsimony where a symmetrical face is easier to comprehend than one complicated. Other signs of our interest in parsimony are our appreciation for poetry (fewer words convey greater meaning) [54], sculpture (elegance and simplicity in design) and our desire for views when buying a house (more stimulation can be received from the same outlook) [55,56]. 

Returning to the example of music, a desire for parsimony may also explain its biological core. Music is a series of noises, but disjointed noise gives no real pleasure. There is structure to music, based on melody, harmony, pitch, rhythm, timbre, pitch, tone, loudness and tempo [57,58,59]. Each style of music varies in the importance and usage of these various characteristics, and can vary with culture [60]. Such variation between the styles makes it difficult to say which feature is at the biological core of musical appreciation. Yet for a single desire there should be one key aspect that is most appealing and common to all variations. There should be a ‘nucleus of common properties’ in music for it to be adaptive [61]. Whether one enjoys jazz, rock and roll, western, oriental or classical is really just a matter of culture and upbringing [62,63]. There is something else. Perhaps the one phrase that can describe what is good about music is that good music is tightly played. In any style of music it is when that style is tightly played by skilled musicians so that it flows and has immaculate or predictable timing that the pleasure is greatest [64,65,66]. The music becomes more musical, and different to noise. This gives the clue as to the character of the desire that appreciates music. 

What the phrase ‘good music is tightly played’ is really describing in biological terms is a desire for parsimony. With parsimony, a simple backbone or foundation occurs, to which all other aspects under consideration are linked, can grow, and return with ease. The music flows but can return in delightful ways back to its central theme, and when played is known to stimulate large-scale cognitive, motor, limbic and reward circuitry in the brain [67]. It is a sign that these adaptations are skilfully and directly tied together, a quality of internal coordination. In a biological sense, it is important for our knowledge and ability to be organised as easily as music. It would be a sign of efficiency in organisation, a pathway to wildness and improved interactive quality, internally and socially. Music is a pleasure because it services what a desire for parsimony is trying to achieve in all aspects of life.

The expanded cerebral cortex in our species accumulates much learning that needs to be sorted into an efficient framework. As a sorting mechanism, a desire for parsimony might be expected to have a relatively greater role in our mind and behavior than in other species. Whether music is simply a spinoff from this overall increased role for parsimony or whether its accomplishment can also be used as an attractive sign to others that the mind is parsimonious and ‘cool’ is difficult to ascertain [68,69]. Music may even assist the continual recasting of the neural network into more parsimonious arrangements, by increasing its plasticity [70].

The reason we appreciate music has remained elusive, and the parsimony theory may go some way to unearthing its underlying character. A core desire for parsimony could link other explanations for musical appreciation. The quality of parsimony increases with attunement due to a broader range or context of information received, while a reciprocal improvement in attunement also occurs because the rules become easier to follow [24]. Therefore, music as an example of parsimony should facilitate improvements in social bonding and cohesion as examples of attunement [14,15,16]. Music may also act as a model of coordination, a play example [10], which inspires or emotionally energizes other areas of activity [9]. Finally, the mastery of music as a symbol of parsimony may make the musician more attractive, and act as a sign that they are capable of a broader set of important life skills. The elegance and ease of parsimony can seem beautiful and attractive. This consequence may give the appearance that music is aimed at sexual selection, like peacock feathers [13], however, it may just be a part of the success that can spin off from being more parsimonious and wild. Not all musical appreciation can be related to sexual tensions and outcomes [8], whereas all could be related to a desire for parsimony as good music is tightly played.

## 3. Possible Mechanism for Parsimony

How can parsimony be pleasurable? If rats, monkeys and other animals have electrodes implanted in their lateral hypothalamus or other ‘pleasure’ regions in the medial forebrain bundle, they will press a lever to stimulate those electrodes above all else, to the point of overriding all other desires such as sex, maternal care and hunger, even causing death through starvation [71,72]. More recently, these regions have been identified as producing desire or want rather than being responsible for the sensation of pleasure. The return feeling of pleasure involves subcortical hotspots such as in the nucleus accumbens, ventral pallidum and brainstem regions such as the parabrachial nucleus in the pons [73,74]. This pleasure reward system seems to have more control over the fundamental desires than it deserves, and suggests that it may be the seat of another strong desire worthy of mention in its own right.

A great deal of research has shown that the pleasure reward system is a stage-gate for sorting bodily needs, producing desires, receiving feedback as pleasure or pain, and eliciting certain reactions based upon that feedback [75]. It can be divided into a wanting stage (desire), liking stage (feeling) and a learning stage that takes note of past experiences so that it can predict future outcomes [74]. The start and finish of this interactive loop appears to be automatic while the cortical assessments made in-between can learn and therefore vary feedback. The hunger desire is produced automatically by the release of the hormone ghrelin from the stomach, which acts upon the hypothalamus and urges the cortex to undertake feeding behaviours [1,2]. While production of the desire is automatic, the experience of how to search for food can be learnt. Similarly, hormones released from the gonads influence the production of sexual desire by the limbic system [76], and learning will then influence how that desire can be met within society. The involvement of the limbic system in producing desires is also demonstrated when damage to certain regions of the hypothalamus can delete the desires for sex, food and water so that the animal must be force fed to keep it alive [77]. At the other end of the interactive loop, the feedback received by the limbic system can also trigger automatic behaviours such as attack, drinking, and feeding and copulatory actions [78].

The limbic system could instill a guide to the learning processes taking place in the cortex if it had a parsimony desire. It could guide and judge interactive success in the cortex by following one simple principle. It must be able to measure the effort of what goes in with what comes out. One of the features of a self-organising system in sensor networks is that the system should have inputs and some measurable output [79]. Neural networks tend to engage in oscillatory activity with each oscillation lasting fractions of a millisecond [80]. The binding or synchronised stimulation of different regions of the brain during each oscillation is thought to enable retrieval and grouping of information from those disparate regions to allow perception and consciousness [81,82,83]. The level of parsimony within each oscillation could be measured. For each pulse of activity that the limbic system sends into the cortex as desire, it should be able to measure the number and breadth of pathways that become stimulated at once and then return that assessment as feeling. Through limbic measurement we could feel pleasure if we gain greater perception and interaction for a given or even reduced neural effort. The more cortical neurons stimulated from the same quantum effort, the greater must be the parsimony and therefore skill of the mind. The pleasure reward system could perform this task as it has connections spread deeply throughout the cortex [72], especially the dorsolateral prefrontal cortex which is the main area for cognition and working memory [74]. 

Some evidence for an interest in parsimony in the brain is that the taste of sugar and the odour of strawberries produce greater pleasure (synergism) than either on its own [84], suggesting that the binding or linking of the two is as important as the individual parts. Also, the hedonic hotspots in the limbic system have been likened to scattered islands that form a single archipelago. ‘Enhancing ‘liking’ above normal by opioid stimulation may require unanimous ‘votes’ in favour from more than one participating hotspot in the forebrain [74,85,86]. This may be the mechanism for measuring parsimony. Improvements in parsimony could stimulate more hotspots at once. ‘True happiness may be a state of liking without wanting’ [74], which is like saying that happiness occurs when a parsimonious mind can achieve pleasure from its base-line inputs of desire (more from less). The efficiency and strength of the parsimony desire could be altered by selecting for the number of hedonic hotspots available and the overall efficiency and distribution of its feedback mechanism.

## 4. Conclusions

Wildness improves survival skills and relevance within environments. The quality of interaction required for wildness is achieved through the parsimonious arrangement of one’s motivations and abilities. With so much to learn, humans have a strong desire for parsimony as shown by our sense for beauty, spirituality, symmetry, elegance, poetry and music. Improvements in parsimony can be experienced during ‘Eureka’ moments and insight. The interactive loop begins as desire in the limbic system, travels through the cortex where it seeks parsimony during learning, and ends as feeling about the outcome in the limbic system. The more pathways that can be stimulated in unison from the same quantum burst of desiring energy the happier we are. That is why we enjoy being right, humour, excitement, insight, imagination, heightened consciousness and awareness. It might also explain our appreciation for music. The parsimony desire feels increased pleasure according to how many ‘hotspot’ pleasure centres can be stimulated at once. The more neurons stimulated at once the greater must be the parsimony and skill of the mind. Our long list of cognitive desires and interests can be related to the important survival need in nature of achieving wildness through parsimony. 
